# Supplementary material for: Copper intrauterine device increases vaginal concentrations of inflammatory anaerobes and depletes lactobacilli compared to hormonal options in a randomized trial
Source: Nat Commun. 2023 Jan 30;14:499. doi: 10.1038/s41467-023-36002-4 (PMC9886933; doi:10.1038/s41467-023-36002-4)
Supplement: Supplementary file 1 — Supplementary Information [file 41467_2023_36002_MOESM1_ESM.pdf]

CONSORT
TRANSPARENT REPORTING of TRIALS

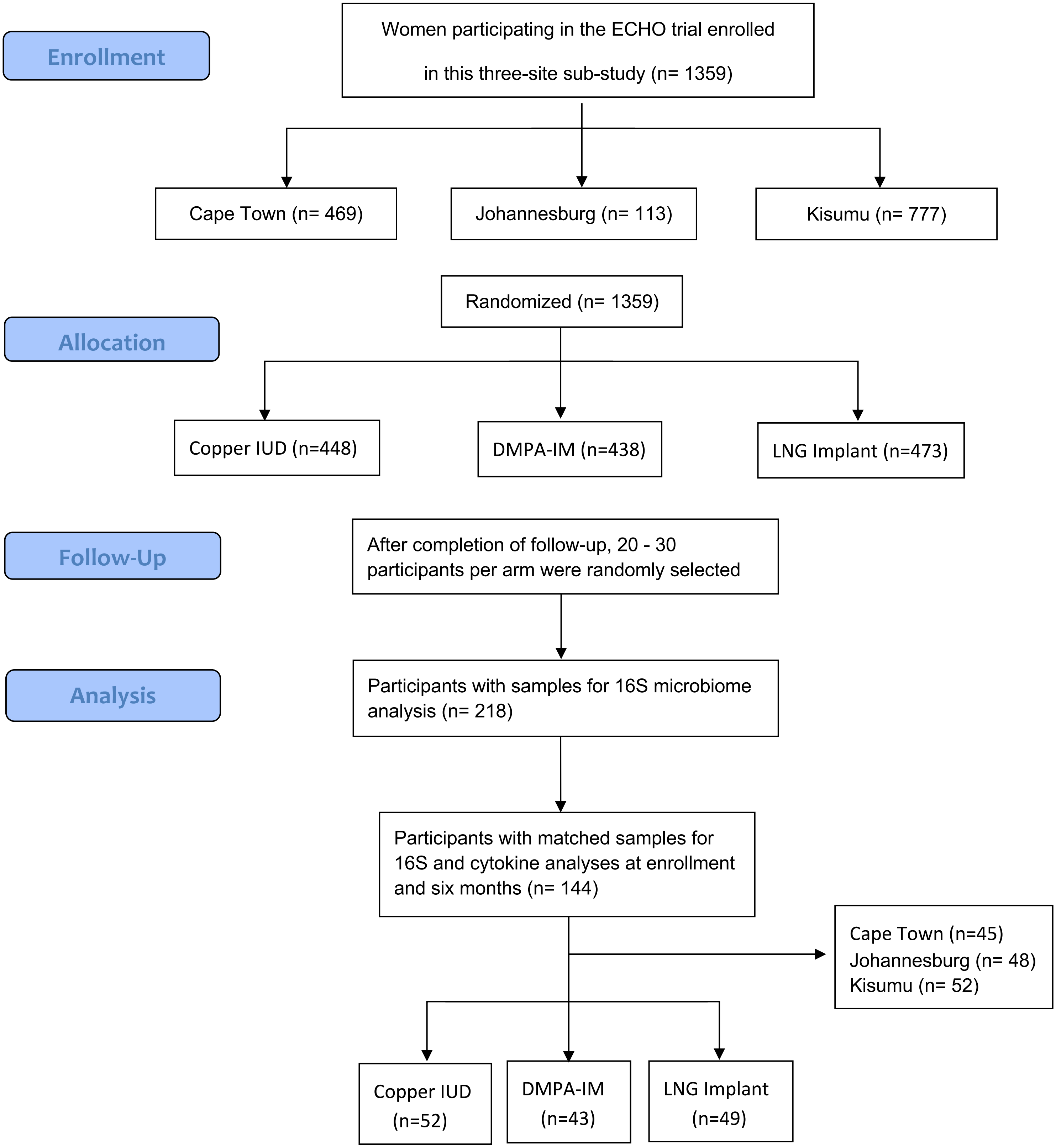

Figure S1. CONSORT flow diagram of participants in this study.

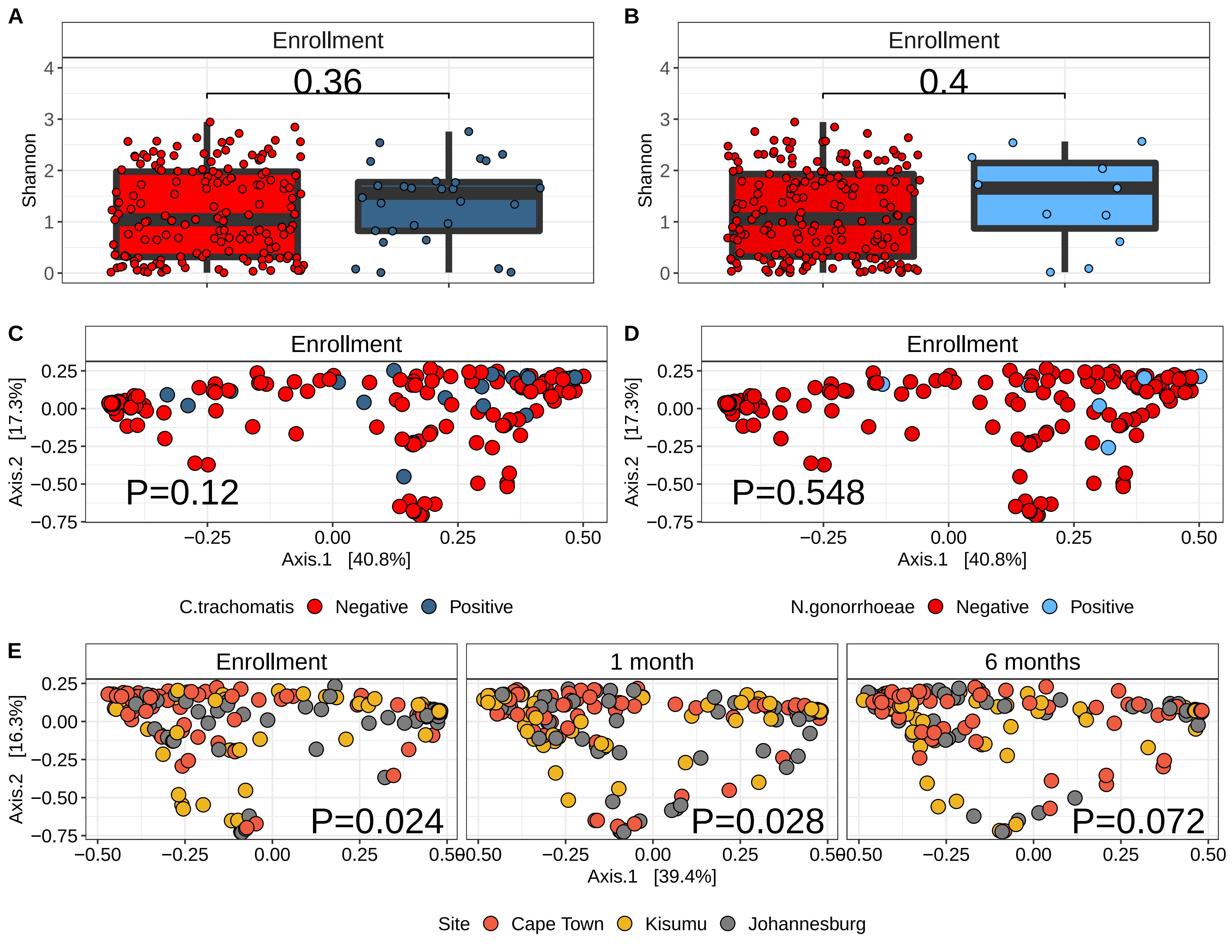
A
Enrollment
0.36
Shannon
B
Enrollment
0.4
C
Enrollment
P=0.12
Axis.2 [17.3%]
Axis.1 [40.8%]
D
Enrollment
P=0.548
C.trachomatis Negative Positive
N.gonorrhoeae Negative Positive
E
Enrollment
P=0.024
1 month
P=0.028
6 months
P=0.072
Axis.2 [16.3%]
Axis.1 [39.4%]
Site Cape Town Kisumu Johannesburg

Figure S2. The effect of STIs and study site on vaginal bacterial profiles. A. Shannon diversity boxplots of samples that tested positive (n=28) or negative (n=165) for *Chlamydia trachomatis*. **B.** Shannon diversity boxplots of samples that tested positive (n=11) or negative (n=182) for *Neisseria gonorrhoeae*. **C.** PCoA ordination (Bray-Curtis distance) of relative abundance transformed bacterial abundance at enrollment, samples are colored by *Chlamydia trachomatis* or **D.** Neisseria *gonorrhoeae* test results. **E**. PCoA ordination (Bray-Curtis distance) of relative abundance transformed bacterial abundance across all timepoints, colored by study site. Boxplot center lines indicate the median, while the hinges indicate the first and third quartiles, and whiskers extend to 1.5 * IQR from the given hinge. Two-tailed P values were calculated with a Wilcoxon Rank Sum test for pairwise comparisons or a PERMANOVA p value from 100 0 permutations for multivariate analysis.

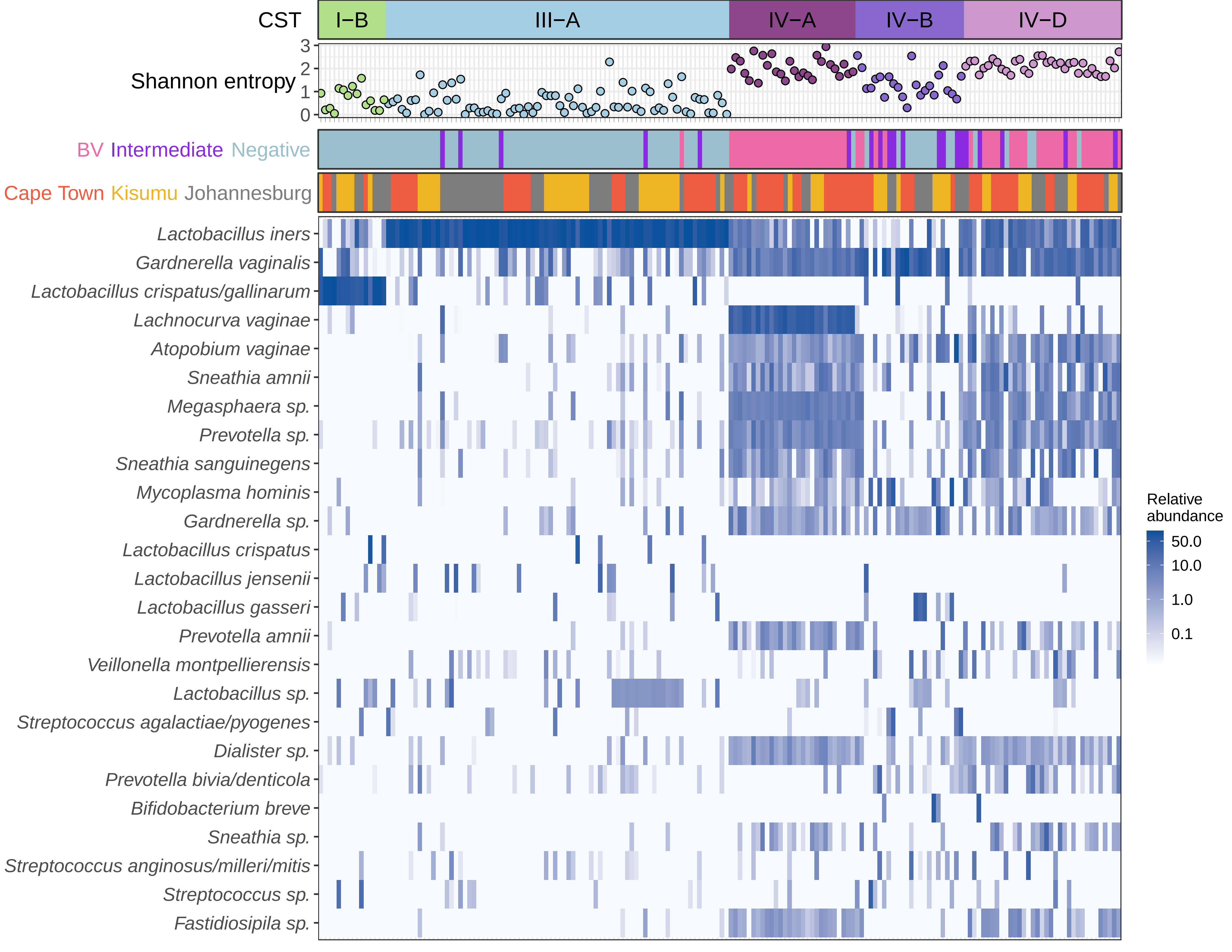
CST
I−B
III−A
IV−A
IV−B
IV−D
Shannon entropy
3
2
1
0
BV Intermediate Negative
Cape Town Kisumu Johannesburg
Lactobacillus iners
Gardnerella vaginalis
Lactobacillus crispatus/gallinarum
Lachnocurva vaginae
Atopobium vaginae
Sneathia amnii
Megasphaera sp.
Prevotella sp.
Sneathia sanguinegens
Mycoplasma hominis
Gardnerella sp.
Lactobacillus crispatus
Lactobacillus jensenii
Lactobacillus gasseri
Prevotella amnii
Veillonella montpellierensis
Lactobacillus sp.
Streptococcus agalactiae/pyogenes
Dialister sp.
Prevotella bivia/denticola
Bifidobacterium breve
Sneathia sp.
Streptococcus anginosus/milleri/mitis
Streptococcus sp.
Fastidiosipila sp.
Relative abundance
50.0
10.0
1.0
0.1

Figure S3. The taxonomic composition, clinical BV status, and distribution across study sites of each CST.

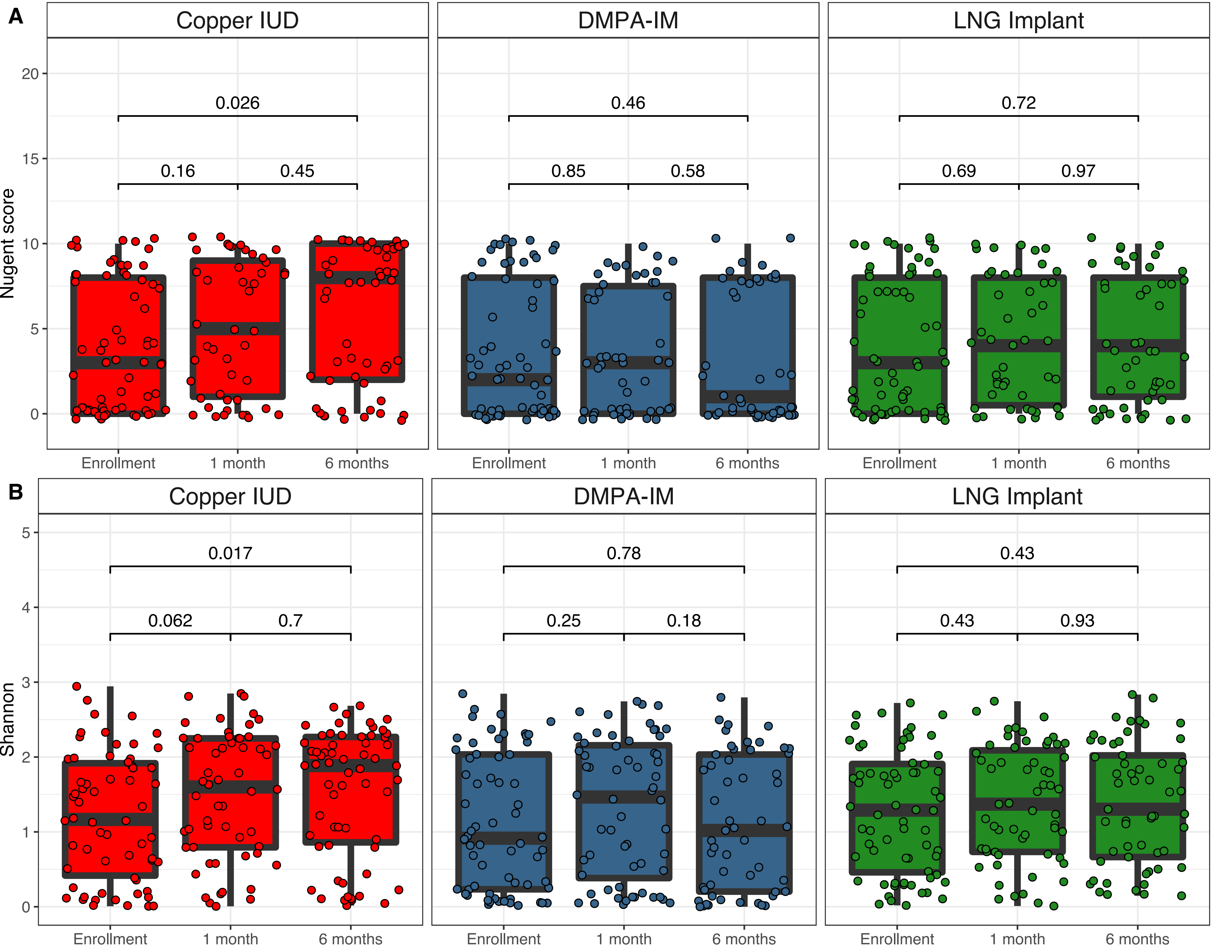
A
Copper IUD
DMPA-IM
LNG Implant
Nugent score
0
5
10
15
20
0.026
0.16
0.45
0.46
0.85
0.58
0.72
0.69
0.97
Enrollment
1 month
6 months
B
Shannon
0
1
2
3
4
5
0.017
0.062
0.7
0.78
0.25
0.18
0.43
0.43
0.93

Figure S4. Contraceptive use significantly alters the clinical and molecular measures of diversity of vaginal bacterial communities across 218 participants. A. Longitudinal Nugent Score boxplots displayed for each contraceptive Cu-IUD (n = 67); DMPA-IM (n = 67); LNG implant (n = 65). **B**. Longitudinal Shannon diversity boxplots stratified by each time point Cu-IUD (n = 70); DMPA-IM (n = 74); LNG implant (n = 74). Boxplot center lines indicate the median, while the hinges indicate the first and third quartiles, and whiskers extend to 1.5 * IQR from the given hinge. Two-tailed P values were calculated using a Wilcoxon Rank Sum test.

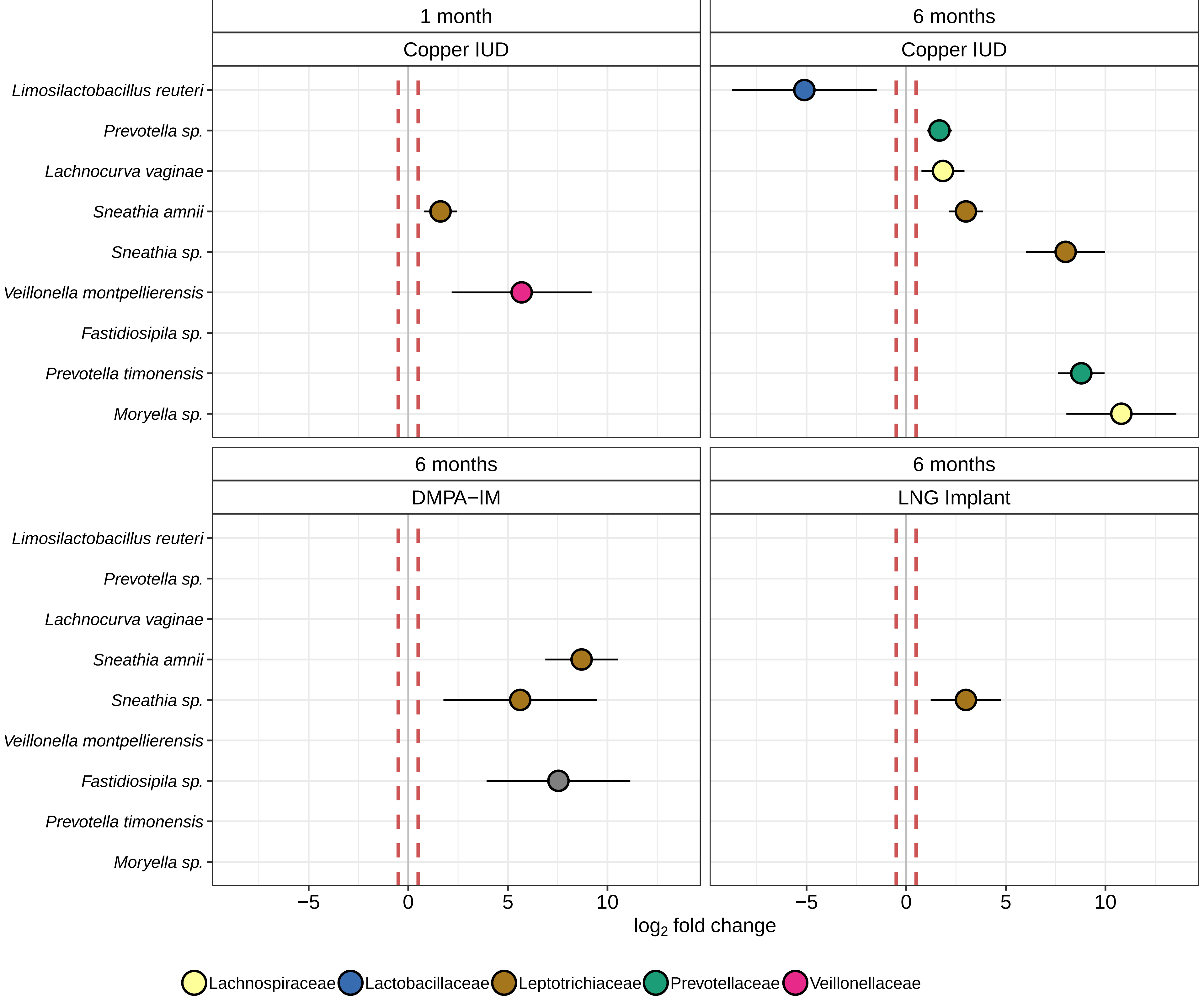
1 month
Copper IUD
6 months
Copper IUD
6 months
DMPA−IM
6 months
LNG Implant
Limosilactobacillus reuteri
Prevotella sp.
Lachnocurva vaginae
Sneathia amnii
Sneathia sp.
Veillonella montpellierensis
Fastidiosipila sp.
Prevotella timonensis
Moryella sp.
−5
0
5
10
log2 fold change
Lachnospiraceae
Lactobacillaceae
Leptotrichiaceae
Prevotellaceae
Veillonellaceae

Figure S5. Fold changes in bacterial abundance after one and six months of contraceptive use, relative to enrollment abundance. Fold changes are $\log_2$ transformed and were calculated using DESeq2. Vertical dashed lines indicate a 0.5 fold change. Each point represents the arithmetic mean $\log_2$ fold change and solid horizontal lines represent the standard error. Taxa with two-tailed Wald P < 0.05 after adjustment for multiple comparisons (Benjamini and Hochberg) are shown; Cu-IUD (n = 55); DMPA-IM (n = 45); LNG implant (n = 53).

A

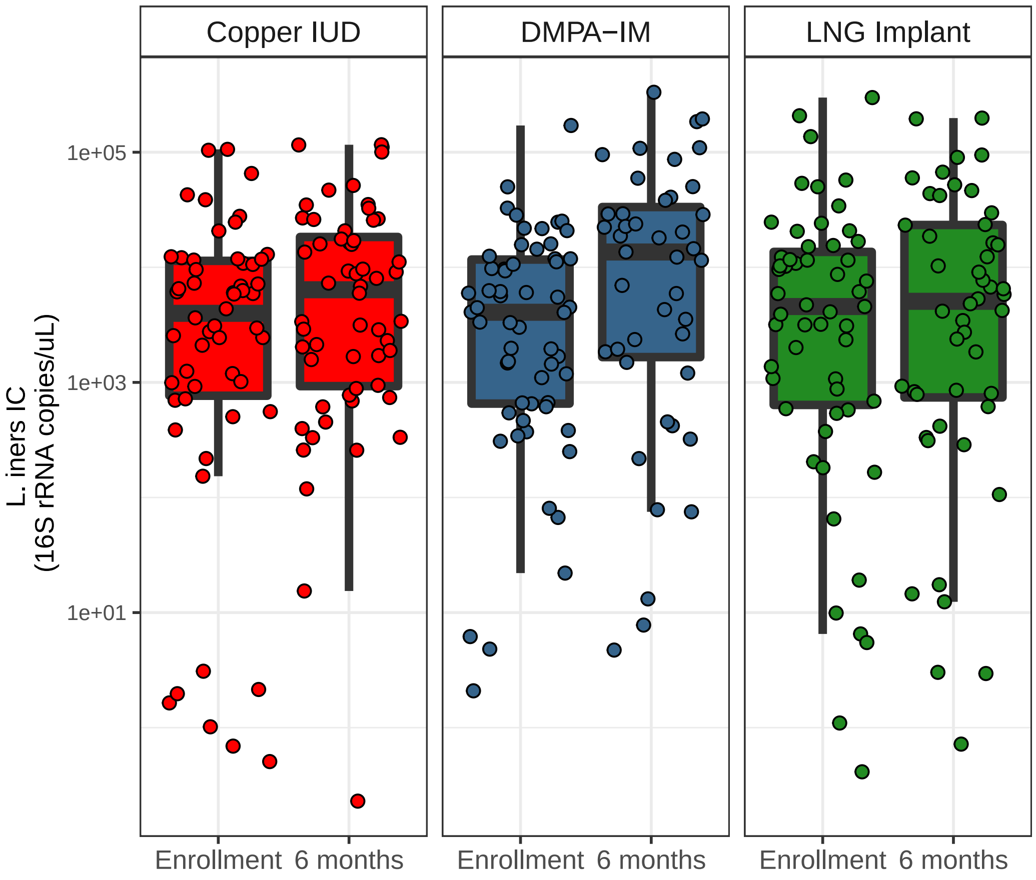
Copper IUD
DMPA−IM
LNG Implant
L. iners IC
(16S rRNA copies/uL)
1e+05
1e+03
1e+01
Enrollment
6 months
Enrollment
6 months
Enrollment
6 months

B

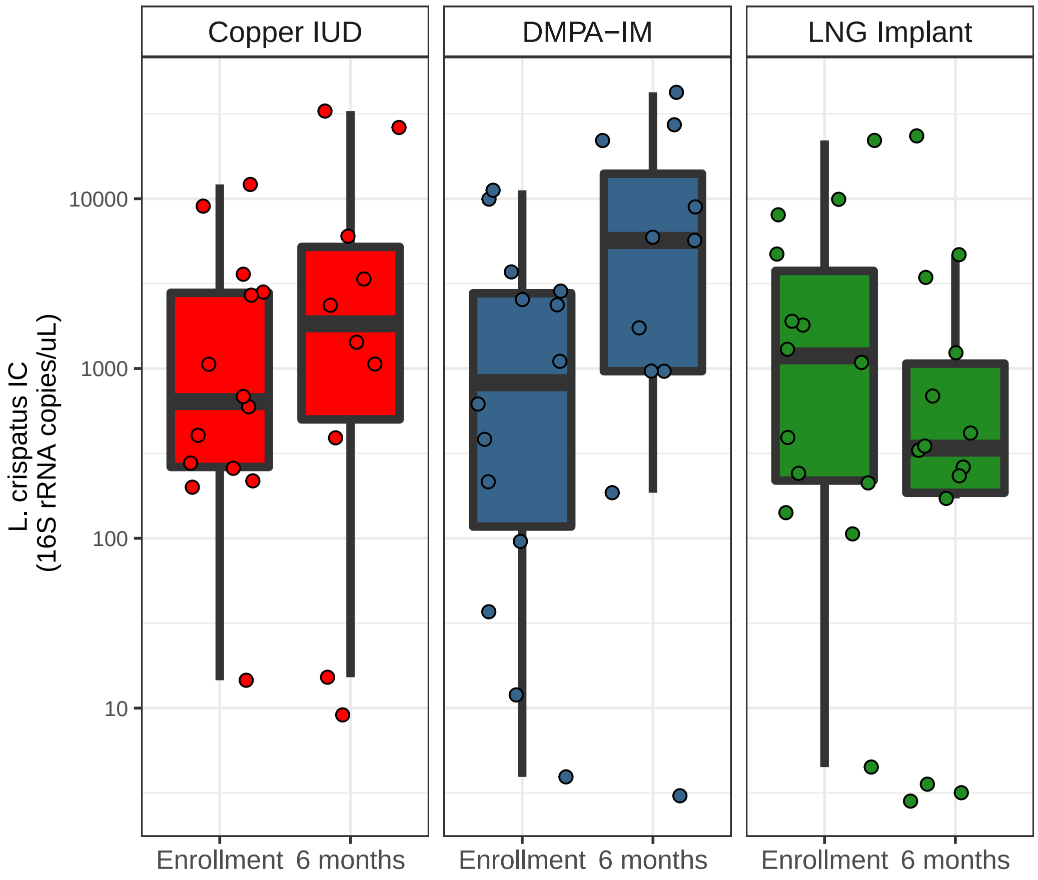
Copper IUD
DMPA−IM
LNG Implant
L. crispatus IC
(16S rRNA copies/uL)
10000
1000
100
10
Enrollment
6 months
Enrollment
6 months
Enrollment
6 months

Figure S6. The effect of contraceptive use on common lactobacilli. Inferred concentrations of **A.** *L. iners* (Cu-IUD (n = 66); DMPA-IM (n = 67); LNG implant (n = 66)) and **B.** *L. crispatus* (Cu-IUD (n = 18); DMPA-IM (n = 23); LNG implant (n = 23)) at enrollment and after six months of contraceptive use. Boxplot center lines indicate the median, while the hinges indicate the first and third quartiles, and whiskers extend to 1.5 * IQR from the given hinge.

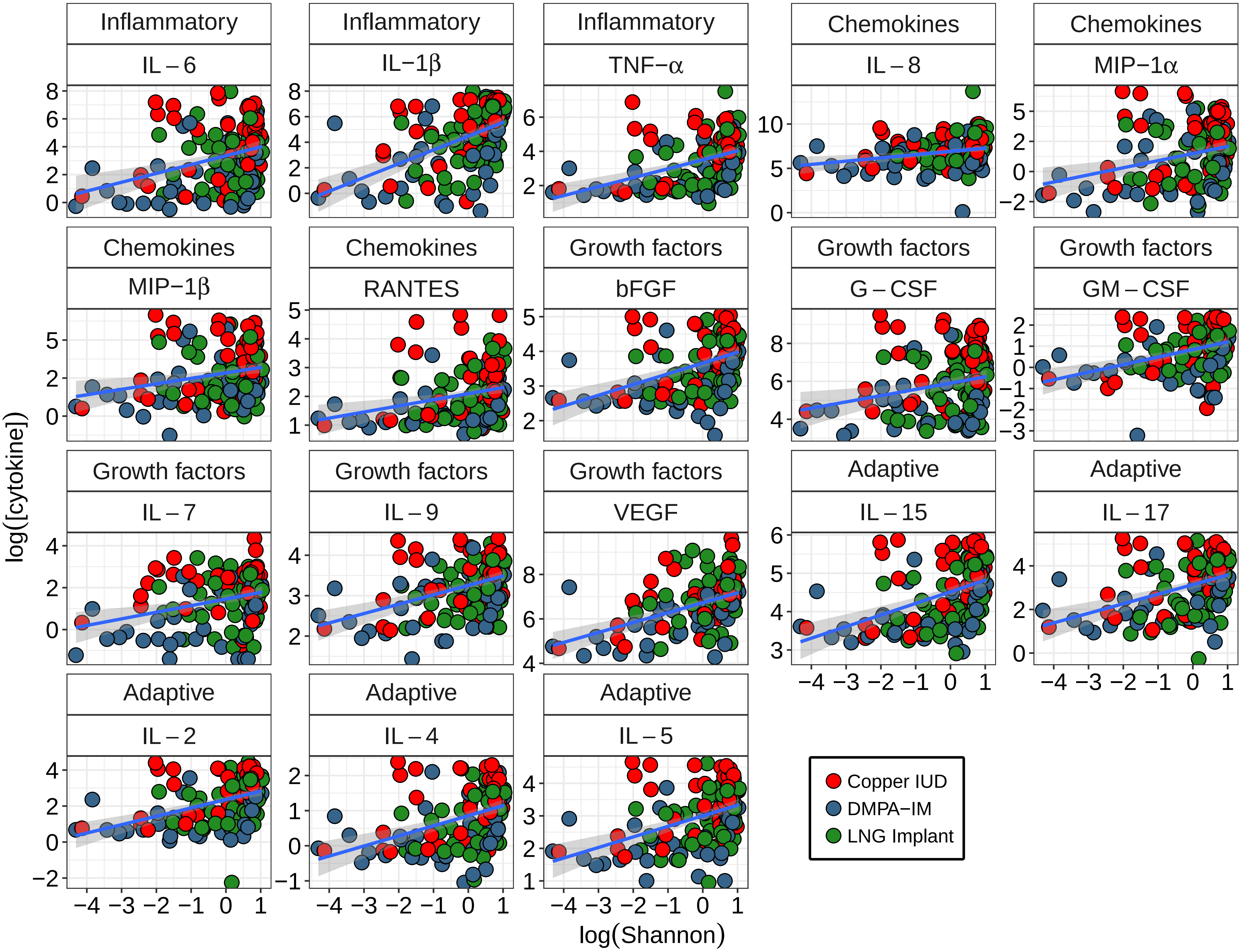

Inflammatory
IL – 6
Inflammatory
IL−1β
Inflammatory
TNF−α
Chemokines
IL – 8
Chemokines
MIP−1α
Chemokines
MIP−1β
Chemokines
RANTES
Growth factors
bFGF
Growth factors
G – CSF
Growth factors
GM – CSF
Growth factors
IL – 7
Growth factors
IL – 9
Growth factors
VEGF
Adaptive
IL – 15
Adaptive
IL – 17
Adaptive
IL – 2
Adaptive
IL – 4
Adaptive
IL – 5
Copper IUD
DMPA−IM
LNG Implant
log([cytokine])
log(Shannon)

Figure S7. Bacterial Shannon index correlates with the abundance of several classes of cytokines after six months of contraceptive use. Cytokine abundance and diversity index values were $\log_e$ transformed prior to model generation. The class of cytokine is indicated above each subplot. Participants are colored by randomization arm. Two-tailed P values were derived from the t-value of the generalized linear model. Data were collected after six months of contraceptive use. Models with $p < 0.05$ after adjustment for multiple comparisons (Benjamini and Yekutieli) are shown, shaded regions around the regression line indicate the 95% confidence interval.

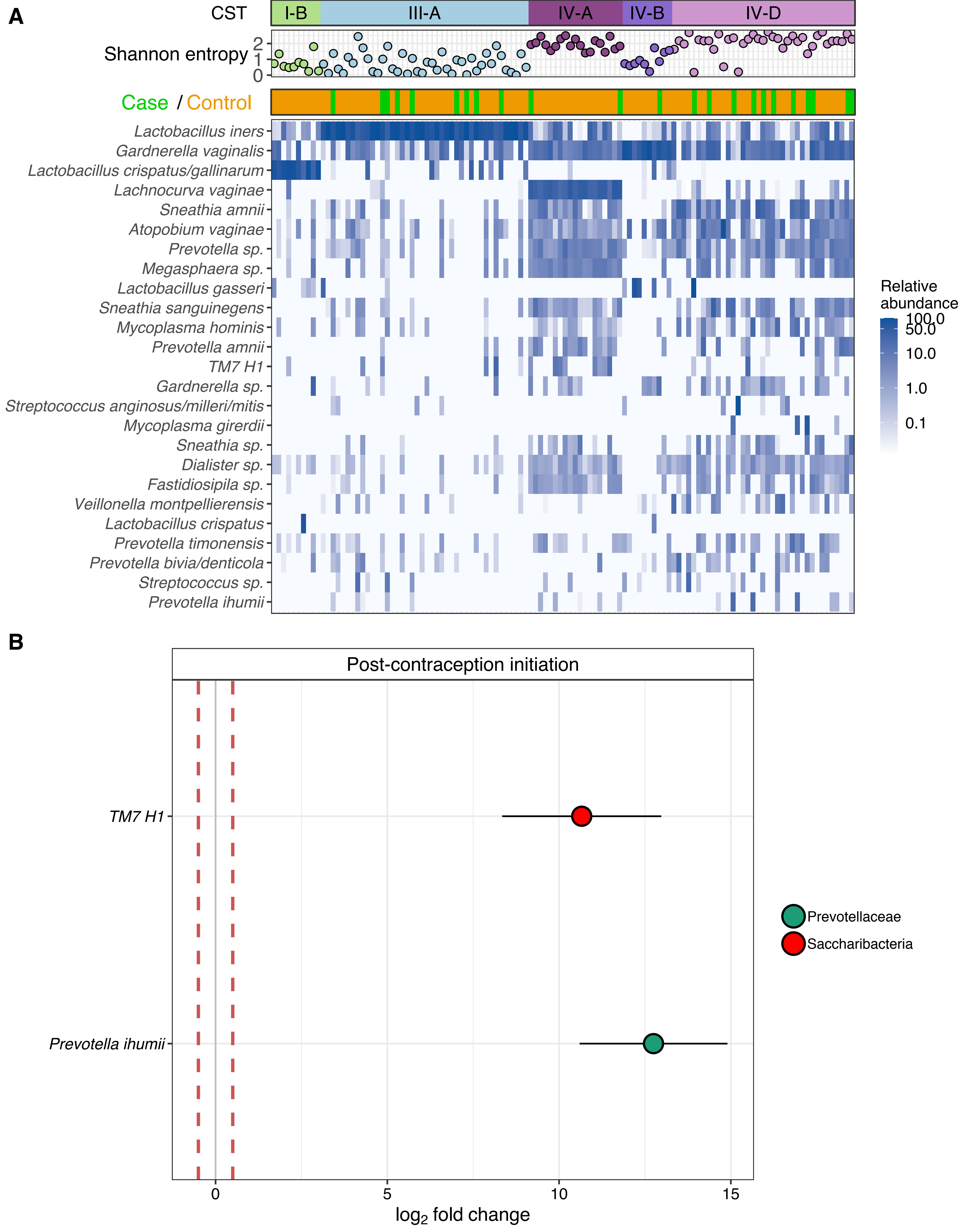

A
CST
I-B
III-A
IV-A
IV-B
IV-D
Shannon entropy
2
1
0
Case / Control
Lactobacillus iners
Gardnerella vaginalis
Lactobacillus crispatus/gallinarum
Lachnocurva vaginae
Sneathia amnii
Atopobium vaginae
Prevotella sp.
Megasphaera sp.
Lactobacillus gasseri
Sneathia sanguinegens
Mycoplasma hominis
Prevotella amnii
TM7 H1
Gardnerella sp.
Streptococcus anginosus/milleri/mitis
Mycoplasma girerdii
Sneathia sp.
Dialister sp.
Fastidiosipila sp.
Veillonella montpellierensis
Lactobacillus crispatus
Prevotella timonensis
Prevotella bivia/denticola
Streptococcus sp.
Prevotella ihumii
Relative abundance
100.0
50.0
10.0
1.0
0.1
B
Post-contraception initiation
TM7 H1
Prevotella ihumii
0
5
10
15
log2 fold change
Prevotellaceae
Saccharibacteria

Figure S8. Bacterial community profiles and shifts in bacterial abundance in cases and controls. A. Relative abundance heatmap of the top 25 taxa across all participants in the case/control analysis (n=118). Community state types are indicated in the top bar. Shannon diversity is displayed in the dotplot and colored by CST. Case/control status is indicated in the middle bar (Case: green; control: orange). **B**. Fold changes in bacterial abundance in case (n=23), relative to control (n=91), participants. Fold changes are $\log_2$ transformed and were calculated using DESeq2. Vertical dashed lines indicate a 0.5 fold change. Each point represents the arithmetic mean $\log_2$ fold change and solid horizontal lines represent the standard error. Taxa with two-tailed Wald $P < 0.05$ after adjustment for multiple comparisons (Benjamini and Hochberg) are shown.

Table S1. Primer constructs targeting the V3-4 region of the 16S rRNA gene used in this study.

Primer name	Index set origin	Index name	Index in sample sheet	Illumina adapter	Index	Frameshift	Linker	Primer sequence - FRAMESHIFTED
F1_MetaIndex	TruSeq i5	D501	TATAGCCT	AATGATACGGCGACCACCGAGATCTACAC	TATAGCCT	G	TCGTCGGCAGCGTC	AATGATACGGCGACCACCGAGATCTACAC TATAGCCT G TCGTCGGCAGCGTC
F2_MetaIndex	TruSeq i5	D502	ATAGAGGC	AATGATACGGCGACCACCGAGATCTACAC	ATAGAGGC	GT	TCGTCGGCAGCGTC	AATGATACGGCGACCACCGAGATCTACAC ATAGAGGC GT TCGTCGGCAGCGTC
F3_MetaIndex	TruSeq i5	D503	CCTATCCT	AATGATACGGCGACCACCGAGATCTACAC	CCTATCCT	CGA	TCGTCGGCAGCGTC	AATGATACGGCGACCACCGAGATCTACAC CCTATCCT CGA TCGTCGGCAGCGTC
F4_MetaIndex	TruSeq i5	D504	GGCTCTGA	AATGATACGGCGACCACCGAGATCTACAC	GGCTCTGA	ATGA	TCGTCGGCAGCGTC	AATGATACGGCGACCACCGAGATCTACAC GGCTCTGA ATGA TCGTCGGCAGCGTC
F5_MetaIndex	TruSeq i5	D505	AGGCGAAG	AATGATACGGCGACCACCGAGATCTACAC	AGGCGAAG	TGCGA	TCGTCGGCAGCGTC	AATGATACGGCGACCACCGAGATCTACAC AGGCGAAG TGCGA TCGTCGGCAGCGTC
F6_MetaIndex	TruSeq i5	D506	TAATCTTA	AATGATACGGCGACCACCGAGATCTACAC	TAATCTTA	CAGTGG	TCGTCGGCAGCGTC	AATGATACGGCGACCACCGAGATCTACAC TAATCTTA CAGTGG TCGTCGGCAGCGTC
F7_MetaIndex	TruSeq i5	D507	CAGGACGT	AATGATACGGCGACCACCGAGATCTACAC	CAGGACGT	CCTGTGG	TCGTCGGCAGCGTC	AATGATACGGCGACCACCGAGATCTACAC CAGGACGT CCTGTGG TCGTCGGCAGCGTC
F8_MetaIndex	TruSeq i5	D508	GTACTGAC	AATGATACGGCGACCACCGAGATCTACAC	GTACTGAC		TCGTCGGCAGCGTC	AATGATACGGCGACCACCGAGATCTACAC GTACTGAC TCGTCGGCAGCGTC
F9_MetaIndex	TruSeq Amplicon	A501	TGAACCTT	AATGATACGGCGACCACCGAGATCTACAC	TGAACCTT	G	TCGTCGGCAGCGTC	AATGATACGGCGACCACCGAGATCTACAC TGAACCTT G TCGTCGGCAGCGTC
F10_MetaIndex	Nextera i5	N501	TAGATCGC	AATGATACGGCGACCACCGAGATCTACAC	TAGATCGC	GT	TCGTCGGCAGCGTC	AATGATACGGCGACCACCGAGATCTACAC TAGATCGC GT TCGTCGGCAGCGTC
F11_MetaIndex	Nextera i5	N502	CTCTCTAT	AATGATACGGCGACCACCGAGATCTACAC	CTCTCTAT	CGA	TCGTCGGCAGCGTC	AATGATACGGCGACCACCGAGATCTACAC CTCTCTAT CGA TCGTCGGCAGCGTC
F12_MetaIndex	Nextera i5	N503	TATCCTCT	AATGATACGGCGACCACCGAGATCTACAC	TATCCTCT	ATGA	TCGTCGGCAGCGTC	AATGATACGGCGACCACCGAGATCTACAC TATCCTCT ATGA TCGTCGGCAGCGTC
F13_MetaIndex	Nextera i5	N504	AGAGTAGA	AATGATACGGCGACCACCGAGATCTACAC	AGAGTAGA	TGCGA	TCGTCGGCAGCGTC	AATGATACGGCGACCACCGAGATCTACAC AGAGTAGA TGCGA TCGTCGGCAGCGTC
F14_MetaIndex	Nextera i5	N505	GTAAGGAG	AATGATACGGCGACCACCGAGATCTACAC	GTAAGGAG	CAGTGG	TCGTCGGCAGCGTC	AATGATACGGCGACCACCGAGATCTACAC GTAAGGAG CAGTGG TCGTCGGCAGCGTC
F15_MetaIndex	Nextera i5	N506	ACTGCATA	AATGATACGGCGACCACCGAGATCTACAC	ACTGCATA	CCTGTGG	TCGTCGGCAGCGTC	AATGATACGGCGACCACCGAGATCTACAC ACTGCATA CCTGTGG TCGTCGGCAGCGTC
F16_MetaIndex	Nextera i5	N507	AAGGAGTA	AATGATACGGCGACCACCGAGATCTACAC	AAGGAGTA		TCGTCGGCAGCGTC	AATGATACGGCGACCACCGAGATCTACAC AAGGAGTA TCGTCGGCAGCGTC
R13_MetaIndex	TruSeq Amplicon	A701	ATCACGAC	CAAGCAGAAGACGGCATACGAGAT	GTCGTGAT	A	GTCTCGTGGGCTCGG	CAAGCAGAAGACGGCATACGAGAT GTCGTGAT A GTCTCGTGGGCTCGG
R14_MetaIndex	TruSeq i7	D701	ATTACTCG	CAAGCAGAAGACGGCATACGAGAT	CGAGTAAT	TC	GTCTCGTGGGCTCGG	CAAGCAGAAGACGGCATACGAGAT CGAGTAAT TC GTCTCGTGGGCTCGG
R15_MetaIndex	TruSeq i7	D702	TCCGGAGA	CAAGCAGAAGACGGCATACGAGAT	TCTCCGGA	CTA	GTCTCGTGGGCTCGG	CAAGCAGAAGACGGCATACGAGAT TCTCCGGA CTA GTCTCGTGGGCTCGG
R16_MetaIndex	TruSeq i7	D703	CGCTCATT	CAAGCAGAAGACGGCATACGAGAT	AATGAGCG	GATA	GTCTCGTGGGCTCGG	CAAGCAGAAGACGGCATACGAGAT AATGAGCG GATA GTCTCGTGGGCTCGG
R17_MetaIndex	TruSeq i7	D704	GAGATTCC	CAAGCAGAAGACGGCATACGAGAT	GGAATCTC	ACTCA	GTCTCGTGGGCTCGG	CAAGCAGAAGACGGCATACGAGAT GGAATCTC ACTCA GTCTCGTGGGCTCGG
R18_MetaIndex	TruSeq i7	D705	ATTCAGAA	CAAGCAGAAGACGGCATACGAGAT	TTCTGAAT	TTCTCT	GTCTCGTGGGCTCGG	CAAGCAGAAGACGGCATACGAGAT TTCTGAAT TTCTCT GTCTCGTGGGCTCGG
R19_MetaIndex	TruSeq i7	D706	GAATTCGT	CAAGCAGAAGACGGCATACGAGAT	ACGAATTC	CACTTCT	GTCTCGTGGGCTCGG	CAAGCAGAAGACGGCATACGAGAT ACGAATTC CACTTCT GTCTCGTGGGCTCGG
R20_MetaIndex	TruSeq i7	D707	CTGAAGCT	CAAGCAGAAGACGGCATACGAGAT	AGCTTCAG		GTCTCGTGGGCTCGG	CAAGCAGAAGACGGCATACGAGAT AGCTTCAG GTCTCGTGGGCTCGG
R21_MetaIndex	TruSeq i7	D708	TAATGCGC	CAAGCAGAAGACGGCATACGAGAT	GCGCATTA	A	GTCTCGTGGGCTCGG	CAAGCAGAAGACGGCATACGAGAT GCGCATTA A GTCTCGTGGGCTCGG
R22_MetaIndex	TruSeq i7	D709	CGGCTATG	CAAGCAGAAGACGGCATACGAGAT	CATAGCCG	TC	GTCTCGTGGGCTCGG	CAAGCAGAAGACGGCATACGAGAT CATAGCCG TC GTCTCGTGGGCTCGG
R23_MetaIndex	TruSeq i7	D710	TCCGCGAA	CAAGCAGAAGACGGCATACGAGAT	TTCGCGGA	CTA	GTCTCGTGGGCTCGG	CAAGCAGAAGACGGCATACGAGAT TTCGCGGA CTA GTCTCGTGGGCTCGG
R24_MetaIndex	TruSeq i7	D711	TCTCGCGC	CAAGCAGAAGACGGCATACGAGAT	GCGCGAGA	GATA	GTCTCGTGGGCTCGG	CAAGCAGAAGACGGCATACGAGAT GCGCGAGA GATA GTCTCGTGGGCTCGG

Primer name	Sequence	Marker gene	Target region
V4_806R_Nextera	GTCTCGTGGGCTCGGAGATGTGTATAAGAGACAGGG**ACTACNVGGGTWTCTAAT**	16S rRNA	V3-V4
V3_357F_Nextera	TCGTCGGCAGCGTCAGATGTGTATAAGAGACAGCC**TACGGGNGGCWGCAG**	16S rRNA	V3-V4

Table S2. Model statistics and prediction accuracy for bacterial inferred concentration-cytokine models at six months of use. Two-tailed P values were derived from the t-value of the generalized linear model. All reported p-values were corrected using the method of Benjamini and Yekutieli.

	Cu-IUD altered bacterial concentrations				
**Dependent Variable**	**Beta Coefficient**	**Standard Error**	**t-statistic**	**adjusted P values[A]**	**MAE[B]**
**IL-1b**	0.284437682	0.057507091	4.946132335	0.000227745	1.653177992
**IL-6**	0.069260858	0.058029524	1.193545177	1	1.863821072
**TNF-a**	0.107664527	0.036351009	2.961802962	0.043723603	1.071315093
**IL-8**	0.059867929	0.044195567	1.354613897	1	1.334564626
**Eotaxin**	0.021097176	0.020241201	1.042288773	1	0.609637209
**IP-10**	-0.179729921	0.058474543	-3.073643857	0.035444947	1.741603382
**MCP-1**	-0.038790204	0.046255676	-0.838604195	1	1.410128288
**MIP-1a**	0.04073915	0.061690302	0.660381762	1	2.030856538
**MIP-1b**	0.002469727	0.046111404	0.053559998	1	1.572466249
**RANTES**	0.026200389	0.024263262	1.079837846	1	0.706788327
**IL-2**	0.105696104	0.029817931	3.544716246	0.013240493	0.897198347
**IL-4**	0.062342787	0.022011828	2.832240321	0.053353902	0.663017303
**IL-5**	0.064939225	0.023027912	2.820022234	0.053353902	0.694607933
**IL-15**	0.071040818	0.020441089	3.475392997	0.013424683	0.607675222
**IL-17**	0.104269615	0.031673895	3.291973286	0.020609517	0.982535341
**IFN-γ**	0.000630958	0.009763007	0.064627453	1	0.226975286
**IL-7**	0.038000216	0.032113913	1.183294468	1	1.001492231
**IL-9**	0.034683665	0.016741953	2.071661845	0.320940762	0.50654532
**bFGF**	0.080274034	0.021184101	3.789352827	0.007430777	0.654816036
**G-CSF**	0.024382315	0.042518225	0.573455626	1	1.387148649
**GM-CSF**	0.072318632	0.027379448	2.641347297	0.080911786	0.742578516
**PDGF-BB**	-0.006811303	0.018578917	-0.366614635	1	0.483318725
**VEGF**	0.120309954	0.026217501	4.588917736	0.000512966	0.732224209
**IL-1Ra**	-0.024743641	0.019766411	-1.251802438	1	0.476912763
**IL-10**	0.019155186	0.024401277	0.785007508	1	0.684711968

[A] P value were adjusted using the method of Benjamini and Yekutieli (2001)

[B]Mean absolute error was calculated from fitting the specified model to a validation dataset, which was a random subset

Distribution of sample counts across the primary and secondary analyses.

**Table S3A.** Sample counts for the primary (pre-post) analysis.			
**Study Site**	**Randomized Arm**	**n***	**n****
Cape Town, South Africa	Cu-IUD	27	24
Cape Town, South Africa	DMPA-IM	28	23
Cape Town, South Africa	LNG Implant	25	21
Kisumu, Kenya	Cu-IUD	20	17
Kisumu, Kenya	DMPA-IM	20	16
Kisumu, Kenya	LNG Implant	20	16
Johannesburg, South Africa	Cu-IUD	20	17
Johannesburg, South Africa	DMPA-IM	20	18
Johannesburg, South Africa	LNG Implant	20	18

*samples selected

**samples that passed QC

**Table S3B.** Sample counts for the secondary (case-control) analysis.		
**Sample Type**	**Study Month**	**n****
Case	1 month	5
Case	3 months	5
Case	6 months	2
Case	9 months	3
Case	12 months	5
Case	15 months	3
Control	Enrollment	4
Control	1 month	17
Control	3 months	19
Control	6 months	11
Control	9 months	14
Control	12 months	18
Control	15 months	12

**samples that passed QC

**Table S3C.** Sample counts that were included in both analyses due to the availability of all primary analysis samples and which were also included in the case-control analysis.		
**Study Site**	**Randomized Arm**	**n****
Cape Town, South Africa	Cu-IUD	5
Cape Town, South Africa	DMPA-IM	12
Cape Town, South Africa	LNG Implant	11
Kisumu, Kenya	Cu-IUD	4
Kisumu, Kenya	DMPA-IM	6
Kisumu, Kenya	LNG Implant	2
Johannesburg, South Africa	Cu-IUD	3
Johannesburg, South Africa	DMPA-IM	2
Johannesburg, South Africa	LNG Implant	2

**samples that passed QC
