# Supplementary material for: Copper intrauterine device increases vaginal concentrations of inflammatory anaerobes and depletes lactobacilli compared to hormonal options in a randomized trial
Source: Nat Commun. 2023 Jan 30;14:499. doi: 10.1038/s41467-023-36002-4 (PMC9886933; doi:10.1038/s41467-023-36002-4)
Supplement: Supplementary file 3 — Reporting Summary [file 41467_2023_36002_MOESM3_ESM.pdf]

nature portfolio

Corresponding author(s): Bryan Brown, Heather Jaspan

Last updated by author(s): Dec 23, 2022

Reporting Summary

Nature Portfolio wishes to improve the reproducibility of the work that we publish. This form provides structure for consistency and transparency in reporting. For further information on Nature Portfolio policies, see our Editorial Policies and the Editorial Policy Checklist.

Statistics

For all statistical analyses, confirm that the following items are present in the figure legend, table legend, main text, or Methods section.

n/a	Confirmed	
☐	☒	The exact sample size (n) for each experimental group/condition, given as a discrete number and unit of measurement
☐	☒	A statement on whether measurements were taken from distinct samples or whether the same sample was measured repeatedly
☐	☒	The statistical test(s) used AND whether they are one- or two-sided<br>*Only common tests should be described solely by name; describe more complex techniques in the Methods section.*
☐	☒	A description of all covariates tested
☐	☒	A description of any assumptions or corrections, such as tests of normality and adjustment for multiple comparisons
☐	☒	A full description of the statistical parameters including central tendency (e.g. means) or other basic estimates (e.g. regression coefficient) AND variation (e.g. standard deviation) or associated estimates of uncertainty (e.g. confidence intervals)
☐	☒	For null hypothesis testing, the test statistic (e.g. F, t, r) with confidence intervals, effect sizes, degrees of freedom and P value noted<br>*Give P values as exact values whenever suitable.*
☒	☐	For Bayesian analysis, information on the choice of priors and Markov chain Monte Carlo settings
☒	☐	For hierarchical and complex designs, identification of the appropriate level for tests and full reporting of outcomes
☐	☒	Estimates of effect sizes (e.g. Cohen's d, Pearson's r), indicating how they were calculated

Our web collection on statistics for biologists contains articles on many of the points above.

Software and code

Policy information about availability of computer code

Data collection	Miseq Reporter Software (2.5) and Bio-plex manager (v4)
Data analysis	Data analysis was conducted using cutadapt (1.16), dada2 (1.12.1), phyloseq (1.38.0), ANCOM-BC (1.4.0), DESeq2 (1.34.0), ashr (2.2-54), cluster (2.1.2), SILVA training set (138), vegan (2.5-7), R (3.6.1). All custom functions and the R code necessary to reproduce the analyses performed here are available at https://github.com/itsmisterbrown/ECHOBioMech.

For manuscripts utilizing custom algorithms or software that are central to the research but not yet described in published literature, software must be made available to editors and reviewers. We strongly encourage code deposition in a community repository (e.g. GitHub). See the Nature Portfolio guidelines for submitting code & software for further information.

Data

Policy information about availability of data

All manuscripts must include a data availability statement. This statement should provide the following information, where applicable:

- Accession codes, unique identifiers, or web links for publicly available datasets
- A description of any restrictions on data availability
- For clinical datasets or third party data, please ensure that the statement adheres to our policy

The datasets generated and/or analyzed during the current study are available at https://doi.org/10.5061/dryad.3n5tb2rmv and the nucleotide sequence data have been deposited in the National Center for Biotechnology Information Sequence Read Archive under accession SUB7926755.

Field-specific reporting

Please select the one below that is the best fit for your research. If you are not sure, read the appropriate sections before making your selection.

- [x] Life sciences
- [ ] Behavioural & social sciences
- [ ] Ecological, evolutionary & environmental sciences

For a reference copy of the document with all sections, see nature.com/documents/nr-reporting-summary-flat.pdf

Life sciences study design

All studies must disclose on these points even when the disclosure is negative.

Sample size

For this substudy, power was estimated as follows:

Randomization arm: Based on previously published data, we estimate a sample size of 30 per group will give 80% power to detect a 50-fold difference in L. jensenii copies/ng between groups (or 90% power to detect 60-fold difference). We note that effect sizes were generally much larger in this study, so this estimate was overconservative. Additionally, for the analysis of changes induced by contraceptive initiation, power calculations were based on mean values of CD4+CD38+ cervical T cells between DMPA and non-DMPA users as reported in previous research (Byrne, 2016). We anticipated that the sample size of 30 subjects in each of the 3 groups will provide 90% power to detect absolute differences in mean frequency of CD4+CD38+ of 1% between any two groups.

HIV seroconversion: Based on the data in the UCHOOSE adolescent cohort (clinicaltrials.gov identifier: NCT02404038) we used a Monte-Carlo simulation procedure (n=1000) with a generalized Wald-type test statistic (as implemented in HMP R package), varying sequencing depth, to estimate that a sample size of 50 women per group (seroconverters and controls) would give ~80% power to detect the estimated differences with 50,000 reads/ sample and a=0.01. We note that effect sizes were generally much larger in this study, so this estimate was overconservative.

Data exclusions

Samples with less than 5,000 filtered, annotated 16S reads were discarded. Cytokines were excluded from this analysis if the inter-assay correlation <0.8 (which included IL-12(p70)). If the concentration of a cytokine fell outside of the limits of detection for a given sample, then that value was not used in statistical analysis. If any cytokine was undetectable in >40% of samples assayed, it was excluded from analyses beyond its initial description.

Replication

Polymerase chain reactions were performed in triplcate. Cytokine specimens from five participants were included across all plates (inter-plate controls). In addition, cytokine samples from five participants were duplicated on each set of plates (intra-plate controls) for quality control measures. Growth curves were assayed in triplicate for each bacteria and copper concentration in each experiment and each experiment was performed three times in total.

Randomization

In this sub-study, which included three of the ECHO Trial sites (Cape Town and Johannesburg, South Africa, and Kisumu, Kenya), we consecutively enrolled all eligible women concurrent to their enrollment in the primary trial, or thereafter if already enrolled (only at Kisumu and Cape Town sites). From those who enrolled in this mucosal sub-study, sample size calculations indicated that 20 participants per randomized arm per site with complete sample sets at all three time points were needed, and thus we randomly selected women who met this criterion at the Johannesburg, South Africa and Kisumu, Kenya sites. However, at the Cape Town, South Africa site, cervical cytobrushes from 80 consecutively enrolled women were processed for phenotyping ex vivo at enrollment and one month post-contraception initiation for a separate study57, and we elected to include all of these participants, rather than a random subset. Additionally, participants that were included in the secondary (case-control) analysis but also had samples available at enrollment, one month, and six months, were added to the pre-post analysis group if not already included. The complete randomized sample counts and sample counts passing QC for the pre-post analysis are available in Table S3A.

For the parent trial randomization procedure, see Ahmed, K., Baeten, J. M., Beksinska, M., Bekker, L. G., Bukusi, E. A., Donnell, D., ... & Welch, J. D. (2019). HIV incidence among women using intramuscular depot medroxyprogesterone acetate, a copper intrauterine device, or a levonorgestrel implant for contraception: a randomised, multicentre, open-label trial. The Lancet, 394(10195), 303-313.

Blinding

Although the Trial was not blinded, all laboratory personnel receiving, processing, and assaying the samples were blinded, and unblinding only occurred at the time of statistical analysis, after a statistical analysis concept sheet had already been formulated with blinded data.

Reporting for specific materials, systems and methods

We require information from authors about some types of materials, experimental systems and methods used in many studies. Here, indicate whether each material, system or method listed is relevant to your study. If you are not sure if a list item applies to your research, read the appropriate section before selecting a response.

Materials & experimental systems

n/a	Involved in the study	
☒	☐	Antibodies
☒	☐	Eukaryotic cell lines
☒	☐	Palaeontology and archaeology
☒	☐	Animals and other organisms
☐	☒	Human research participants
☐	☒	Clinical data
☒	☐	Dual use research of concern

Methods

n/a	Involved in the study	
☒	☐	ChIP-seq
☒	☐	Flow cytometry
☒	☐	MRI-based neuroimaging

Human research participants

Policy information about studies involving human research participants

Population characteristics	See Table 1; In this sub-study, which included three of the ECHO Trial sites (Cape Town and Johannesburg, South Africa, and Kisumu, Kenya), we consecutively enrolled all eligible women concurrent to their enrollment in the primary trial. From those who enrolled in this mucosal sub-study, 20-30 women per contraceptive arm per site were randomly selected for analyses of samples collected at enrollment (pre-contraceptive initiation), one month, and six months post-contraceptive initiation.
Recruitment	Eligibility and randomization procedure for the parent trial is described in detail elsewhere (see Ahmed et al. 2019), but importantly included women seeking effective contraception and being in the age range of 16-35 years. Notably, all participants reported no contraceptive use during the six months preceding enrollment and initiated the assigned contraceptive after the enrollment samples were collected. In this sub-study, which included three of the ECHO Trial sites (Cape Town and Johannesburg, South Africa, and Kisumu, Kenya), we consecutively enrolled all eligible women concurrent to their enrollment in the primary trial, or thereafter if already enrolled (only at Kisumu and Cape Town sites). From those who enrolled in this mucosal sub-study, sample size calculations indicated that 20 participants per randomized arm per site with complete sample sets at all three time points were needed, and thus we randomly selected women who met this criterion at the Johannesburg, South Africa and Kisumu, Kenya sites. However, at the Cape Town, South Africa site, cervical cytobrushes from 80 consecutively enrolled women were processed for phenotyping ex vivo at enrollment and one month post-contraception initiation for a separate study, and we elected to include all of these participants, rather than a random subset. Additionally, participants that were included in the secondary (case-control) analysis but also had samples available at enrollment, one month, and six months, were added to the pre-post analysis group if not already included. The complete randomized sample counts and sample counts passing QC for the pre-post analysis are available in Table S3A.
Ethics oversight	The protocol was approved by the Human Research Ethics Committee of the University of Washington (STUDY00000261), Kenya Medical Research Institute Scientific and Ethics Review Unit (SERU/CMR/P0014/3109), University of Witwatersrand Human Research Ethics Committee (HREC PRC 141112), University of Cape Town Human Research Ethics Committee (HREC 371/2015), and FHI360 (523201). Women provided written informed consent and remuneration of participants was done in accordance with the requirements of local ethics committees to provide fair compensation without inducement.

Note that full information on the approval of the study protocol must also be provided in the manuscript.

Clinical data

Policy information about clinical studies

All manuscripts should comply with the ICMJEguidelines for publication of clinical research and a completedCONSORT checklist must be included with all submissions.

Clinical trial registration	NCT02550067
Study protocol	https://clinicaltrials.gov/ct2/show/NCT02550067
Data collection	Eligibility and randomization procedure for the parent trial is described in detail elsewhere (Ahmed 2019), but importantly included women seeking effective contraception and being in the age range of 16-35 years. In this sub-study, which included three of the ECHO Trial sites (Cape Town and Johannesburg, South Africa, and Kisumu, Kenya), we consecutively enrolled all eligible women concurrent to their enrollment in the primary trial. From those who enrolled in this mucosal sub-study, 20-30 women per contraceptive arm per site were randomly selected for analyses of samples collected at enrollment (pre-contraceptive initiation), one month, and six months post-contraceptive initiation. For the case-control analysis, age and site matched controls were chosen from the enrollment, one- and six month visits as in the pre-post analysis, as well as from nine-, twelve-, and fifteen- month visits, depending upon when the matched case tested positive during the study.
Outcomes	The primary endpoint of the parent trial was incident HIV infection, identified using a standard seroconversion algorithm, occurring after enrolment. For women testing HIV seropositive, we assessed archived plasma samples from the enrolment visit using HIV RNA PCR and excluded those with detectable HIV RNA. Secondary outcomes were pregnancy, serious adverse events, adverse events resulting in method discontinuation, and method continuation. The primary safety endpoint of the study was defined as any serious adverse event or any adverse event resulting in method discontinuation, until the trial exit visit at 18 months. For more detail, see Ahmed, K., Baeten, J. M., Beksinska, M., Bekker, L. G., Bukusi, E. A., Donnell, D., ... & Welch, J. D. (2019). HIV incidence among women using intramuscular depot medroxyprogesterone acetate, a copper intrauterine device, or a levonorgestrel implant for contraception: a randomised, multicentre, open-label trial. The Lancet, 394(10195), 303-313.

The primary endpoints for this mucosal substudy were changes in Th17 cell frequency, and microbial diversity induced by hormonal contraceptive initiation. Secondary outcomes were these changes associated with later HIV seroconversion.
